# Household profiles of neglected tropical disease symptoms among children: A latent class analysis of built-environment features of Tanzanian households using the Demographic and Health Survey

**DOI:** 10.7189/jogh.12.04067

**Published:** 2022-09-03

**Authors:** Francisco A Montiel Ishino, Claire Rowan, Charlotte Talham, Kevin Villalobos, Dikshit Poudel, Janani Rajbhandari-Thapa, Joel Seme Ambikile, Faustine Williams

**Affiliations:** 1 Division of Intramural Research, National Institute on Minority Health and Health Disparities, National Institutes of Health, Bethesda, MD, USA; 2 Department of Epidemiology, Rollins School of Public Health, Emory University, Atlanta, Georgia, USA; 3 Department of Agricultural and Applied Economics, College of Agricultural and Environmental Science, University of Georgia, Athens, Georgia, USA; 4 Department of Health Policy and Management, College of Public Health, University of Georgia, Athens, Georgia, USA; 5 Department of Community Health Nursing, Muhimbili University of Health and Allied Sciences, Dar es Salaam, United Republic of Tanzania

## Abstract

**Background:**

While malaria and neglected tropical disease (NTD) morbidity and mortality rates among children <5 years old have decreased through public health efforts in the United Republic of Tanzania, associations between household environments and disease outcomes are relatively unknown.

**Methods:**

We conducted latent class analysis (LCA) on 2015-2016 Tanzania Demographic Health Survey data from mothers with children <5 years old (N = 10 233) to identify NTD household risk profiles. The outcome of child NTD was assessed by mothers’ reports of recent diarrhoea, cough, treatment for enteric parasites, and fever symptoms. Household-built environment indicators included urban/rural designation, electricity access, water source, cooking fuel, flooring, wall, and roofing materials. External environmental covariates were considered to further differentiate profiles.

**Results:**

Five profiles were identified in the sample: rural finished walls households (40.2%) with the lowest NTD risk; rural rudimentary households (20.9%) with intermediate-low NTD risk; finished material households (22.5%) with intermediate NTD risk; urban households (14.4%) with intermediate-high NTD risk and high likelihood of enteric parasites; rural finished roof/walls households (2.1%) with the highest overall NTD risk.

**Conclusions:**

This study is among the first to use LCA to examine household environment characteristics to assess child NTD risk in Tanzania. This paper serves as a framework for community-level rapid NTD risk assessment for targeted health promotion interventions.

Over a billion people are impacted by neglected tropical diseases (NTDs) globally [1]. Based on the 2010 Global Burden of Disease Study, an estimated 48 million disability-adjusted life years (DALYs) have been lost to NTDs annually [2] and over 350 000 lives [3]. Much of the burden of NTDs falls on children. Globally, childhood NTDs have both a drastic impact on child health affecting physical and cognitive development and on the global burden of child morbidity and mortality [4]. In the United Republic of Tanzania (henceforth Tanzania), preventable and treatable diseases take the lives of 270 children <5 years of age every day [5]. Childhood mortality in Tanzania varies by geographic region, ranging from 56 per 1000 live births in the north to 88 per 1000 live births in the Lake regions, and by urban-rural divisions, from 87 per 1000 live births in urban areas to 76 per 1000 live births in rural areas [5].

Tanzania is endemic to five NTDs (i.e., onchocerciasis, lymphatic filariasis, soil-transmitted helminths, schistosomiasis, and trachoma) for which preventative chemotherapy is administered [6]. Due to the Tanzanian government’s efforts to take an integrated approach to controlling NTDs, coordination units across the country reached 53% geographic coverage as of 2011 [7]. Additionally, Tanzania has an extensive database and monitoring system for tracking progress towards the management and elimination of several NTDs [7]. Still, there are many structural and logistic difficulties to eliminating NTDs in Tanzania and the surrounding areas. Although mass drug administration programmes are widely seen as cost-efficient and effective methods for reducing NTD morbidity and mortality, mass drug administration programmes are often implemented by health ministries in countries with endemic NTDs with heavy reliance on schools to administer treatment to children [8]. As a result, preschool-age children and children who do not attend school tend to be overlooked, putting them at increased risk of disease.

The impact of housing construction on the risk of vector-borne diseases is well understood [9]. In Africa, increasing insecticide resistance has raised concerns about the future efficacy of bed nets [3]. Considering this, targeted, location-specific approaches are necessary to eliminate NTDs across the globe. For instance, Yé et al. [10] found a higher risk of contracting malaria in mud-roofed homes compared to homes with iron-sheet roofs in Burkina Faso. Similar studies have also found poor housing construction to increase presence of mosquitos in the household as well as malaria risk [11-13]. Additionally, substandard housing environments (including poor sanitation infrastructure) increase the risk of NTDs and NTD symptoms, particularly for children [4,14,15]. Compared to adults, children face greater exposure to microbes during play and are far more susceptible to environmental exposures in and outside the home [4]. In a study based in the coastal regions of Tanzania, Armah et al. [16] found that good housing quality was negatively associated with NTD risk. Further, they found highly localized geographic differences in NTD risk, raising concerns that such disparities run the risk of being overlooked when monitoring the burden of NTDs at regional or national levels [16]. To address the unique geographic and environmental factors afflicting impoverished children and families, developing systems to rapidly assess NTD risk in localized areas to direct interventions to the most highly burdened communities is crucial. Our study follows these established risks by holistically examining the environmental contexts of the household in which children <5 years old experience NTD symptoms.

NTDs are exacerbated by syndemic interactions with each other, the “big three” (i.e., malaria, tuberculosis, and HIV), and the socio-environmental contexts in which they occur [17]. In this study, we adopt a syndemic perspective for our person-centred analysis of anthropogenic environmental factors that exist at the household level to perpetuate the burden of NTDs in children <5 years old. To accomplish this, we used latent class analysis (LCA) to examine associations between household infrastructures and children <5 years old’s NTD symptoms in Tanzania. This study contributes to existing global health literature on household-built environment risk factors for NTDs. Additionally, our study offers a framework for rapid risk assessment of childhood NTDs to address the need for localized assessments and tailored interventions for the most vulnerable and often neglected neighbourhoods and households plagued by disproportionate morbidity.

## METHODS

The Demographic and Health Surveys (DHS) Program primarily funded by United States Agency for International Development (USAID) is an international program that supports a range of tailored data collection options in host countries. DHS has been providing technical assistance to more than 90 countries on more than 300 surveys since 1984 [18]. The Tanzania Demographic Health Survey (TZ-DHS) was designed to provide data by monitoring the population and its health through interviews and surveys with local women. The 2015-2016 survey cycle is the sixth DHS Survey conducted in Tanzania since the first one in 1991-1992. Approximately 13 360 households were selected through a stratified random sample to represent the urban and rural areas and 12 563 were successfully interviewed [19]. Our analysis used data from the 2015-2016 survey cycle of mothers with children <5 years (N = 10 233). The DHS Program and Institutional Review Board assessed the research protocol. The DHS Program approved the protocol and released the necessary data for analysis. The Institutional Review Board deemed that no further ethical approval was necessary as no human participants were involved in this study. Data can be made available by inquiring with the DHS Program.

### Latent class analysis

#### Household built-environmental indicators

We conducted LCA, an approach that allows the identification of otherwise unidentified/unobservable subgroups or profiles. Observable environmental household factors were used as indicators for NTD symptoms. Factors included the location of residence (rural/urban), access to electricity (yes/no), location of household water source (in dwelling/in yard or plot/elsewhere), type of cooking fuel used in the home (electricity/bottled fuel/solid fuel or other), and type of material used for roofing, flooring, and walls (natural or rudimentary materials (e.g., grasses, mud)/finished materials (e.g., fired brick, cement)).

#### Distal outcome: neglected tropical disease symptoms

Our primary outcome of interest was NTD symptoms in children <5 years old. Clinically assessed NTD symptoms were reported by mothers using binary responses (yes/no) to the following: 1) drugs for intestinal parasites in the last six months; 2) report of cough within last two weeks; 3) report of diarrhoea within last two weeks; and 4) fever indicating a sign/symptom of malaria two weeks prior to survey. While malaria is not an NTD, malarial fever was included as malaria is over-diagnosed in Tanzania (often without confirmation), overlooking possible NTD-related symptoms [20].

#### Covariates to assess latent class membership

**Figure 1:**
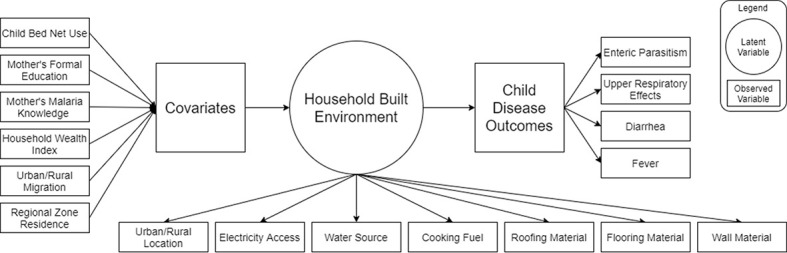
Latent class analysis model framework.

Several covariates external to the household and not included in the LCA were considered to assess and differentiate identified latent classes: the use of bed net by children during the previous night (yes/no), the mother receiving formal education (yes/no), knowledge of malaria prevention (yes/no), household wealth index (poorest or poorer/middle, wealthier, or wealthiest), migration of the family from the countryside (yes/no) or town (yes/no), and regional data collection zones. The regional zones included Central Tanzania (i.e., Dodoma and Singida), Coastal Tanzania (i.e., Dar es Salaam, Tanga, Morogoro and Pwani), Northern Highlands of Tanzania (i.e., Arusha and Kilimanjaro; Western Tanzania: Katavi and Kigoma), Lake Region (i.e., Mwanza, Shinyanga, Kagera, Mara, Manyara, Simiyu, Geita and Tabora), Southern Highlands of Tanzania (i.e., Iringa, Mbeya, Rukwa and Njombe), Southern Tanzania (i.e., Lindi, Mtwara and Ruvuma), and Zanzibar Island of Tanzania (ie, Kaskazini (Kaz.) and Kusini (Kus.), Unguja, Mjini Magharibi, and Kaz. and Kus. Pemba). **Figure 1** shows the model and data analysis framework, as well as illustrates how the constructs between covariates, household indicators, and NTD symptoms among children <5 years are interrelated.

#### Latent class analysis and model fit criteria

All analyses accounted for the TZ-DHS 2015-16 complex survey design by applying sample weights. All models were weighted and accounted for household clustering and stratification. A comparative model fit approach was used to determine the number of classes. Multiple models were created (ie, 1- to 7-class solutions) to select the best model with: (1) Bayesian information criterion (BIC); (2) sample-size-adjusted Bayesian information criterion (SSA-BIC); and (3) high entropy (ie, the acceptable quality of classification) [21]. Models were also assessed on their practical and theoretical implications. Multivariate logistic regression was used on external household factors not included in the LCA to better define class memberships of the five-class solution selected for interpretation. All LCAs were conducted using Mplus version 8.4 (Muthén & Muthén, Los Angeles CA, USA). All code used in our analytical procedure can be made available by request.

## RESULTS

Most households were in rural areas (72.9%), did not have electricity (83.2%), did not have a water source on the property (94.2%), cooked using a heat source other than electricity or bottled fuel (97.6%), had natural or rudimentary flooring (64.8%), and had finished walls (76.6%) and roofs (70.0%). Additionally, most households reported no enteric parasites within the last six months (65.7%), cough in the last two weeks (83.8%), or diarrhoea in the last two weeks (87.9%) in children under five. However, 87.7% of households reported malaria-related fever in children under five. See Table S1 in the **Online Supplementary Document** for further details.

### Latent class analysis: Household characteristics

**Figure 2:**
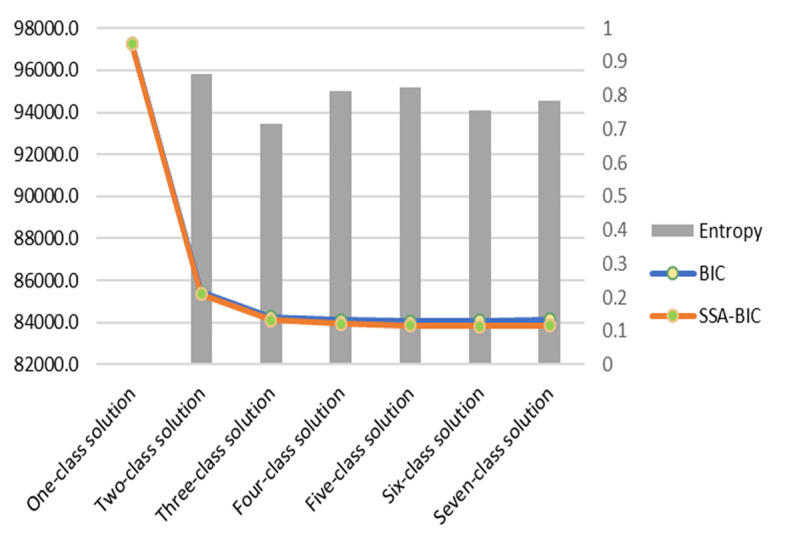
Latent class model fit criteria comparison.

The LCA yielded a five-class solution with an entropy of 0.82 (see **Figure 2**; Table S2 in the **Online Supplementary Document** has the complete model fit criteria used for comparisons to assess the best model solution for interpretation). Each class was assigned a label based on the highest conditional probabilities of environmental household factors within that class. The Class 1 profile, hereafter referred to as rural finished wall households, represented 40.2% of the sample. These households had the highest conditional probabilities of being in a rural area (94.2%), being without electricity (100.0%), and sourcing water from outside the dwelling or yard (98.0%) compared to all other classes. Rural finished wall households exclusively used cooking fuel other than electricity or bottled sources (100.0%). Furthermore, these households had the highest probabilities of having natural or rudimentary flooring (99.8%) and finished walls (100.0%) compared to other classes. They also had high conditional probabilities of both natural or rudimentary (41.8%) and finished (58.2%) roofs.

Class 2, or rural rudimentary households, represented 20.9% of the sample. These homes were largely rural (94.1%), were without electricity (99.5%), sourced water from outside the dwelling or yard (96.0%), and exclusively used fuel other than electricity or bottled sources (100.0%). Rural rudimentary households had a high conditional probability of having natural or rudimentary floors (98.4%) and the highest conditional probabilities of natural or rudimentary walls (97.6%) and roofs (65.2%) compared to other classes.

Class 3 (22.5%), referred to as finished material households, consisted of households with high conditional probabilities of having finished flooring (78.2%), walls (94.2%) and roofs (99.7%). These households also had high conditional probabilities of not having electricity (89.1%), sourcing water from outside the dwelling or yard (90.0%) and using fuel other than electricity or bottled sources (99.5%). Finished material households had 38.4% conditional probabilities of being in urban regions and 61.6% of being in rural regions.

The Class 4 profile (14.4%), or urban households, had the highest conditional probabilities of being in an urban region (92.0%), having electricity (93.3%), and having a personal water source in the dwelling (7.1%) and in the yard/plot (15.1%) compared to all other classes. Still, urban households had the greatest conditional probability of sourcing water from outside of the home or yard (77.8%). Compared to other groups, urban households also had the highest conditional probabilities of having finished flooring (99.2%), walls (99.4%), and roofs (99.9%).

**Figure 3:**
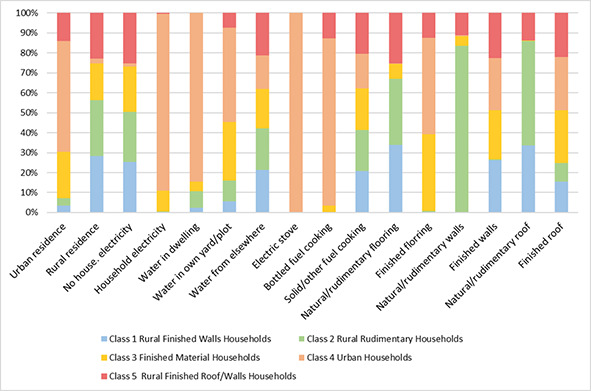
Distribution of conditional probabilities from the five-class solution by household characteristics.

The Class 5 profile (2.1%), or rural finished roof/walls households, was characterized by households with high conditional probabilities of being in a rural area (76.9%), being without electricity (99.5%), having the water source outside of dwelling or yard (97.6%), and using fuel other than electricity or bottled sources (98.1%). These households had rudimentary flooring (74.7%) but had high conditional probabilities of having finished walls (87.0%) and roofs (82.9%). See **Figure 3** for the distribution of conditional probabilities. Table S3 in the **Online Supplementary Document** has the complete household-built environment conditional probabilities for each class.

### Latent class analysis: Neglected tropical disease risk

**Figure 4:**
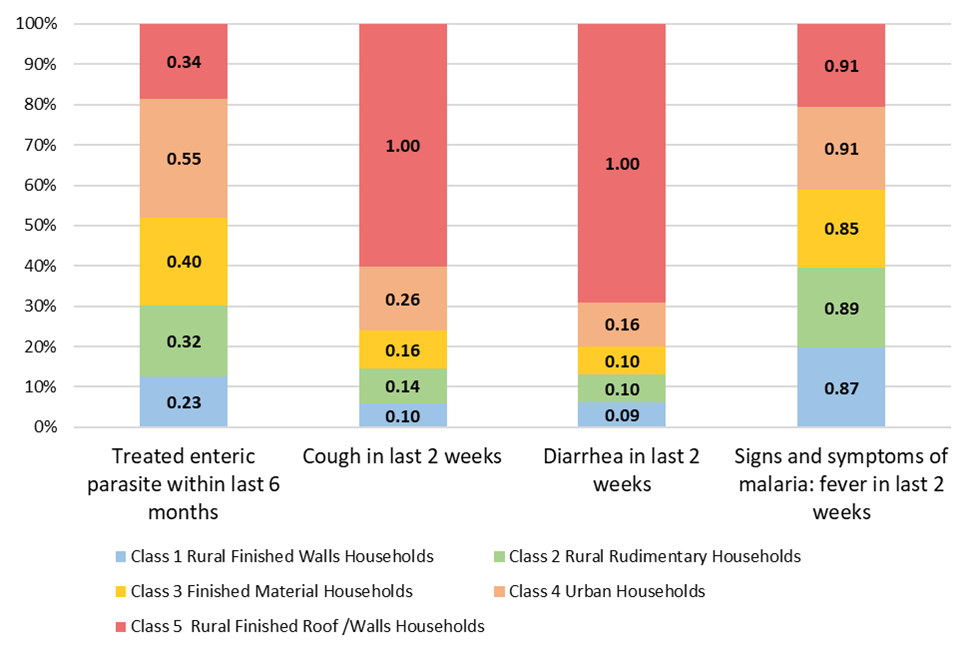
Stacked bar graph represents proportions of the conditional probabilities of child-under-5 NTD disease outcomes by classes named by household characteristics. Numbers within bar segments represent conditional probabilities from the latent class analysis of the presence of the NTD symptom.

Rural finished wall households had the lowest reports of treating enteric parasites in the last 6 months (23.4%) or cough (9.7%) and diarrhoea (9.1%) in the last two weeks. These households had an 87.2% likelihood of reporting fever as a sign or symptom of malaria. Rural rudimentary households had a likelihood of 32.3% of treating a child under 5 for enteric parasites in the last 6 months and likelihoods of 14.3% and 9.7% of reporting cough and diarrhoea in the last 2 weeks, respectively. These households also had a high conditional probability of fever as a sign or symptom of malaria (88.6%). Finished material households had the second-highest conditional probability of enteric parasites (40.3%) and the lowest probability of fever as a sign or symptom of malaria (85.2%) compared to all other classes. These households had a likelihood of cough and diarrhoea in the last two weeks of 15.8% and 10.3%, respectively. Urban households had the highest conditional probabilities of having treated a child <5 years for enteric parasites (54.5%) and fever as a sign or symptom of malaria (91.2%) compared to other classes. Additionally, these households had moderate conditional probabilities of cough (26.2%) and diarrhoea (15.8%) in the last 2 weeks. Rural finished roof and walls households had conditional probabilities of 100.0% of reporting each cough and diarrhoea in children <5 years in the last two weeks and 90.0% of reporting fever as a sign or symptom of malaria. Additionally, these households had a 34.1% likelihood of treating enteric parasites in the last 6 months. See **Table 1** for complete conditional probabilities of NTD symptoms, and **Figure 4** for the visual distribution of conditional probabilities. Table S4 in the **Online Supplementary Document** has all covariate comparisons between classes.

**Table 1:** Distal outcomes of 5-class solution

	Class 1	Class 2	Class 3	Class 4	Class 5
	**Rural finished walls households**	**Rural rudimentary households**	**Finished material households**	**Urban households**	**Rural finished roof/walls households**
	**Lowest risk**	**Intermediate-low risk**	**Intermediate risk**	**Intermediate-high risk (parasite risk)**	**Highest risk**
	40.2%	20.9%	22.5%	14.4%	2.1%
	N = 4111	N = 2134	N = 2302	N = 1469	N = 216
**Treated enteric parasite within last 6 months**					
No	0.766	0.677	0.597	0.455	0.659
Yes	0.234	0.323	0.403	0.545	0.341
**Cough in last 2 weeks**					
No	0.903	0.857	0.842	0.738	0.000
Yes	0.097	0.143	0.158	0.262	1.000
**Diarrhoea in last 2 weeks**					
No	0.909	0.903	0.897	0.842	0.000
Yes	0.091	0.097	0.103	0.158	1.000
**Signs and symptoms of malaria: fever in last 2 weeks**					
No	0.128	0.114	0.148	0.088	0.091
Yes	0.872	0.886	0.852	0.912	0.909

## DISCUSSION

We used a novel mixture model approach to identify unique heterogeneous profiles of households based on their built environmental features and reports of NTD symptoms observed in children <5 years. Household infrastructure characteristics and child-under-five NTD symptom histories were assessed through interviews and surveys of Tanzanian mothers. Our analysis yielded five household profiles characterized by infrastructural factors: rural finished walls, rural rudimentary, finished material, urban, and rural finished roof/walls households. Along with unique features that defined the household groups, distinct NTD symptom profiles were observed in each class. The built environment classes we identified have the potential to serve as a rapid risk assessment framework for NTDs among children aged <5 years. Our findings contribute to the emerging literature on the impact of NTDs on impoverished regions and can be used to create more targeted approaches to public health interventions in Tanzania and the surrounding areas.

Rural finished roof/walls households were identified as the highest risk profile for childhood NTD symptoms overall. Households in this category had a high probability of malaria-related fever and all reported cough and diarrhoea in the last two weeks in a child under five. Conversely, rural finished wall households revealed the lowest risk of NTD symptoms including low reports of cough and diarrhoea in children under five. Compared to rural finished roof/walls households, rural finished wall households had higher conditional probabilities of being in rural areas, having natural or rudimentary flooring and roofing, and having finished walling. The lower risk of NTD symptoms in rural finished wall households could partially be explained by a decreased entry of mosquitoes and other disease-carrying vectors [22,23]. Further research is needed to identify additional factors that contribute to the disparities in NTD symptoms between these two household profiles.

Rural rudimentary and finished material households had very similar NTD risk profiles. Rural rudimentary households had slightly higher conditional probabilities of enteric parasites, cough, and diarrhoea in children under five and a slightly lower probability of malaria-related fever, consistent with prior literature [22-24]. These households were differentiable by the types of materials used for flooring, walls, and roofs and by their rural-urban classifications. A greater proportion of finished material households were in urban residences compared to rural rudimentary households which may account for the higher conditional probability in these households of receiving treatment for enteric parasites.

Urban households were additionally found to have the highest conditional probability of treating enteric parasites in a child under five compared to all other classes. Due to rapidly growing urban areas in low- and middle-income countries, in-dwelling sanitation facilities and sewerage may not be available nor possible [25]. The use of public sanitation facilities may explain the higher likelihood of enteric parasite reports in high-population urban and pe-urban areas with poor water, sanitation, and hygiene conditions [26]. The identified urban households, however, also had the highest conditional probabilities of having a water source in the dwelling and yard or plot when compared to other household profiles. In some cases, soil helminth reinfections are also possible, especially among children in urban settings with poor water, sanitation, and hygiene conditions [27,28]. This may also be indicative of the urban and rural healthcare divide; while urban areas have more access to healthcare facilities and resources, this does not translate to quality and availability of care [29].

The conditional probabilities of malaria-related fever ranged from 85.2% to 91.2% across all household groups. The high conditional probabilities of malaria across profiles could be due to the high prevalence of malaria in Tanzania but may also be attributable to misdiagnoses of the cause of fever due to the perceived impact of malaria [30,31]. Compared to other household profiles, urban households had the highest probability of malaria-related fever. Interestingly, the multivariate logistic regression revealed that compared to urban households, rural rudimentary, finished material, rural finished roof/walls, and rural finished walls households all had lower odds of having knowledge of malaria. Additionally, all but rural finished roof/walls households had lower odds than urban households of children sleeping under a bed net. Governmental and other relevant non-governmental organizations should prioritize malarial fever awareness and environmental sanitation in areas with endemic malaria [24].

By using a mixture model approach, we were able to account for the multiple interactions that may be NTD and NTD-related comorbidities. For instance, in relation to malarial overdiagnosis symptoms of fever and fatigue will often lead to a self-diagnosis of malaria [30-32]. This self-attribution of disease could include a constellation of other coinfections from schistosomiasis, helminthiases, or visceral leishmaniasis [33-36]. In many cases, some NTDs may predispose others to malaria or vice versa as is the case with schistosomiasis [33,36]. Moreover, symptoms like diarrhoea are not unique to ascariasis, schistosomiasis, and/or chagas, which may also have cough and fever as symptoms as well. Respiratory and gastrointestinal symptoms may be occurring in parallel with other diseases and interacting with other symptoms and diseases that could be indicative of an NTD, multiple NTDs, or concurrent infections [2,33,35,36]. The distal outcomes for our analysis, however, are not mutually exclusive and are cooccurring with the given conditional probabilities to the observed outcome. As such, the purpose of this work was to explore risk factors for symptoms that could be attributable to NTDs, but not exclusive to NTDs due to the secondary nature of datasets like the DHS.

Our findings have the potential to be used as a risk assessment tool for identifying households with children at risk for NTDs. These findings can also assist in the implementation of tailored health interventions. However, investigative fieldwork is needed in Tanzania to evaluate whether our identified household profiles can be confirmed in urban and rural communities. Moreover, as urban centres in Tanzania continue to grow, pre-urban areas are critical in understanding NTD risk in the context of household and environmental factors. Future studies can leverage cellular network access, as networks have expanded throughout the African continent, increasing the potential to use mobile devices for healthcare delivery and risk assessments [7]. Digital infrastructures could be used in field research to evaluate the existence of the profiles identified in this paper, as well as deliver interventional health services. The distribution of bed nets, indoor residual spraying, and impregnated bed sheets by community-based interventions has been shown to be effective at reducing parasitaemia and malaria incidence and prevalence [24,37]. Additionally, sanitation services and facilities must be focused to avoid the health effects of continued parasitic infection on children such as developmental stunting, wasting, and deficits [38,39], as well as diminished efficacy of vaccines and other prophylactic treatments [40,41]. These types of interventions could similarly be effective in reducing NTD risk through educational efforts, health promotions, and distribution of household construction resources. Finally, the use of secondary data that is cross-sectional in nature, is not causal bur could lead to more detailed and confirmatory studies that could be based on symptomology as well as confirm possible NTDs given the co-occurring likelihood of symptoms given the disease.

To our knowledge, this is the first study to conduct LCA on household factors to identify child subgroups at high risk of preventable diseases in Tanzania. It has several limitations. Data used in our analysis was cross-sectional and self-reported by participants. Therefore, they do not capture the temporal nature of NTD risks and are susceptible to bias. Additionally, data are not based on diagnostic test results, as such we do not know the NTD status of children under five in our analysis. Furthermore, our study included mother’s educational level as a proxy for socioeconomic status. However, future analyses should incorporate social determinants of health, including sociodemographic and socioeconomic indicators, to formulate a more robust model. Ethnicity may be an important factor affecting health outcomes and receipt of health care services in Tanzania [16]. Existing disease risk assessment tools primarily rely on spatial analysis of environmental exposures or epidemiological surveys [42,43]. These analyses overlook nuanced risk at the community and household levels. The benefit of our analysis is that it combined built environment exposures with epidemiological data to propose a rapid risk assessment framework designed for household level surveillance in order to design the most cost-effective and equitable NTD interventions.

## CONCLUSIONS

Our analysis identified five household-built environment profiles and their respective disease outcomes for children <5 years using latent class analysis, a person-centred approach for finding unobservable profiles of possible risk. Initial findings indicate a high prevalence of malaria-related fever in both rural and urban households and more frequent treatment of enteric parasites in urban areas. Further analysis is needed to confirm the presence of the hypothesized risk groups in the surveyed population and to measure the perceived and actual prevalence of neglected tropical disease symptoms in the region. Our approach expands the possibilities of quantitative models to rapidly assess child-under-five disease risk based on interacting contextual factors. The results from this study can be used to improve neglected tropical disease interventions and help reduce child-under-five morbidity and mortality in Tanzania.

## Additional material


Online Supplementary Document
