# Supplementary material for: Household profiles of neglected tropical disease symptoms among children: A latent class analysis of built-environment features of Tanzanian households using the Demographic and Health Survey
Source: J Glob Health. 2022 Sep 3;12:04067. doi: 10.7189/jogh.12.04067 (PMC9441010; doi:10.7189/jogh.12.04067)
Supplement: Online Supplementary Document [file jogh-12-04067-s001.pdf]

Online Supplementary Document

Table S1. Household sample descriptives.

	N	%
Type of residence		
Urban	2,776	27.1%
Rural	7,456	72.9%
Electricity in household		
No	8,119	83.2%
Yes	1,642	16.8%
Household source of water		
In own dwelling	72	0.9%
In own yard/plot	400	5.0%
Elsewhere	7,604	94.2%
Type of cooking fuel		
Electricity	34	0.4%
Bottled gas/paraffin/kerosene	201	2.1%
Solid fuel or other source	9,523	97.6%
Household flooring		
Natural or rudimentary	6,330	64.8%
Finished	3,432	35.2%
Household walls		
Natural or rudimentary	2,281	23.4%
Finished	7,481	76.6%
Household roof		
Natural or rudimentary	2,930	30.0%
Finished	6,832	70.0%
Treated enteric parasite within last 6 months		
No	6,244	65.7%
Yes	3,259	34.3%
Cough in last 2 weeks		
No	7,949	83.8%
Yes	1,539	16.2%
Diarrhea in last 2 weeks		
No	8,293	87.9%
Yes	1,142	12.1%
Signs and symptoms of malaria: fever		
No	1,178	12.3%
Yes	8,385	87.7%

Table S2. Latent class model fit comparisons.

Models	BIC	ssa-BIC	Entropy
One-class solution	97,276.7	97,235.4	-
Two-class solution	85,447.9	85,362.1	0.862
Three-class solution	84,249.1	84,118.8	0.714
Four-class solution	84,103.8	83,929.0	0.814
Five-class solution	84,063.2	83,843.9	0.824
Six-class solution	84,100.1	83,836.4	0.755
Seven-class solution	84,153.9	83,845.6	0.786

Notes. BIC = Bayesian information criterion
ssa-BIC = sample size adjusted Bayesian information criterion

Table S3. Conditional probabilities of infrastructural characteristics from 5-class solution model.

	Class 1	Class 2	Class 3	Class 4	Class 5
	**Rural Finished Walls Households**	**Rural Rudimentary Households**	**Finished Material Households**	**Urban Households**	**Rural Finished Roof/Walls Households**
	40.2% *N*=4,111	20.9% *N*=2,134	22.5% *N*=2,302	14.4% *N*=1,469	2.1% *N*=216
Type of residence					
Urban	0.058	0.059	0.384	0.920	0.231
Rural	0.942	0.941	0.616	0.080	0.769
Electricity in household					
No	1.000	0.995	0.891	0.067	0.995
Yes	0.000	0.005	0.109	0.933	0.005
Household source of water					
In own dwelling	0.002	0.007	0.004	0.071	0.000
In own yard/plot	0.018	0.033	0.095	0.151	0.024
Elsewhere	0.980	0.960	0.900	0.778	0.976
Type of cooking fuel					
Electricity	0.000	0.000	0.000	0.023	0.000
Bottled fuels	0.000	0.000	0.005	0.127	0.019
Solid fuel or other	1.000	1.000	0.995	0.849	0.981
Household flooring					
Natural or rudimentary	0.998	0.984	0.218	0.008	0.747
Finished	0.002	0.016	0.782	0.992	0.253
Household walls					
Natural or rudimentary	0.000	0.976	0.058	0.006	0.130
Finished	1.000	0.024	0.942	0.994	0.870
Household roof					
Natural or rudimentary	0.418	0.652	0.003	0.001	0.171
Finished	0.582	0.348	0.997	0.999	0.829

Table S4. Multinomial logistic regression of covariates to assess profile membership using Class 4 Urban Households as reference.

	Class 1			Class 2			Class 3			Class 5		
	Rural Finished Walls Households			Rural Rudimentary Households			Finished Material Households			Rural Finished Roof/Walls Households		
		95% CI			95% CI			95% CI			95% CI	
Covariates	OR	Lower	Upper	OR	Lower	Upper	OR	Lower	Upper	OR	Lower	Upper
Child slept under bed net	**0.29**	**0.15**	**0.58**	**0.26**	**0.13**	**0.50**	**0.49**	**0.27**	**0.88**	0.58	0.19	1.75
Middle/Wealthier/Wealthiest*	**0.00**	**0.00**	**0.16**	**0.00**	**0.00**	**0.10**	**0.00**	**0.00**	**0.34**	**0.00**	**0.00**	**0.44**
Knows ways to avoid malaria	**0.00**	**0.00**	**0.09**	**0.00**	**0.00**	**0.07**	**0.01**	**0.00**	**0.17**	**0.00**	**0.00**	**0.07**
Mother w/ no formal education	**21.1**	**5.96**	**74.7**	**24.5**	**7.02**	**85.2**	**8.77**	**2.55**	**30.2**	**15.1**	**1.27**	**179.7**
Moved from town	2.66	0.58	12.2	0.48	0.20	1.15	1.24	0.63	2.41	0.62	0.13	3.04
Moved from countryside	**37.5**	**9.73**	**144.4**	**7.32**	**3.53**	**15.2**	**5.39**	**2.98**	**9.75**	2.54	0.75	8.65
Central†	2.11	0.66	6.75	0.80	0.23	2.77	0.71	0.27	1.89	0.42	0.00	295.9
Coastal†	**0.00**	**0.00**	**0.04**	**0.03**	**0.01**	**0.10**	**0.07**	**0.03**	**0.17**	**0.01**	**0.00**	**0.15**
Northern Highlands†	0.80	0.24	2.64	1.98	0.71	5.52	1.10	0.50	2.39	0.96	0.18	5.22
West†	1.13	0.09	13.6	0.53	0.05	6.15	0.39	0.04	4.16	1.02	0.05	20.55
Lake†	1.35	0.56	3.27	0.55	0.20	1.52	0.81	0.36	1.80	1.06	0.20	5.78
Southern Highlands†	**0.19**	**0.06**	**0.62**	**0.03**	**0.01**	**0.12**	**0.37**	**0.15**	**0.90**	**0.05**	**0.00**	**0.69**

Notes: CI = confidence interval
OR = odds ratio
*poorest/poorer as reference
†Zanzibar as reference
